# Coping strategies and health literacy among Jordanian patients with myocardial infarction

**DOI:** 10.4102/curationis.v49i1.2835

**Published:** 2026-10-22

**Authors:** Deyaaldeen M. Alrababah, Penelope Martin, Pat M. Mayers

**Affiliations:** 1 School of Nursing, Faculty of Community and Health Sciences, University of the Western Cape, Cape Town, South Africa

**Keywords:** coping strategies, health literacy, myocardial infarction, patients, Jordan

## Abstract

**Background:**

Patients with myocardial infarction require adaptive coping skills and adequate health literacy levels to improve their health and reduce morbidity, cost of care and mortality.

**Objectives:**

This study examined the association between commonly used coping strategies and health literacy levels among Jordanian patients with myocardial infarction.

**Method:**

A descriptive cross-sectional survey design was used. Systematic random sampling was used to recruit 140 patients with myocardial infarction from four hospitals in Jordan. Respondents completed two scales: the Arabic versions of the Brief COPE (AR-BC) and the 16 items European Health Literacy Survey Questionnaire (HLS-EU-Q16). The research process followed ethical principles and measures.

**Results:**

Respondents had low scores of engaging in coping activities. Emotion-focused coping and religious strategies were most used. Less than half (39.3%, *n* = 55) had adequate health literacy. The highest score was for the dimension Applying Information and the lowest was for Information Processing. Positive associations were found between health literacy levels and positive coping strategies (planning, acceptance, active coping, emotional support and positive reframing) and negative associations between negative coping strategies (venting) and health literacy levels.

**Conclusion:**

Patients with myocardial infarction had low health literacy and made more use of emotion-focused strategies rather than problem-focused coping strategies. Higher health literacy levels may be associated with the use of more adaptive coping strategies. Health literacy assessment and patients’ use of adaptive coping strategies should be fundamental to the nursing care of patients with myocardial infarction.

**Contribution:**

Jordanian patients post-myocardial infarction have limited health literacy and use mainly emotion-focused coping strategies. To promote patients’ adaptive, problem-focused coping, we recommend that health literacy training is included in nursing education curricula and HL screening is included in cardiac rehabilitation programmes.

## Introduction

Myocardial infarction causes more deaths than any other disease or illness (World Health Organization [WHO] 2020). It is an unexpected life-threatening event, necessitating adaptive coping to prevent recurrence, improve health and reduce mortality (Blumenthal et al. 2016; Hussain, Khalily & Ahmad 2020; Lynggaard et al. 2017). After controlling for socioeconomic and health status, maladaptive coping strategies have been reported to be associated with a shorter lifespan (Trudel-Fitzgerald et al. 2022). Patients with myocardial infarction require an adequate health literacy level to understand health information and utilise adaptive coping strategies (Peltzer et al. 2020). Inadequate health literacy levels have been associated with patients’ use of maladaptive coping strategies (Peltzer et al. 2020). These lead to higher mortality rates, readmissions, poor quality of life (De Melo Ghisi et al. 2018; Kanejima et al. 2022; Peltzer et al. 2020) and increased costs of care (Palumbo 2017). Patients with low health literacy levels are estimated to have a 62.1% higher mortality risk and an 18.4% increased risk of rehospitalisation (Kanejima et al. 2022). Coping is a continuous process of cognitive and behavioural changes to manage demands that are appraised by individuals as exceeding their resources (Folkman & Lazarus 1984:141). Coping strategies have been categorised into emotion-focused and problem-focused. Emotion-focused coping, primarily used if the individual feels nothing can be performed (Folkman & Lazarus 1984), includes emotional support, venting, humour, acceptance, religious coping, self-blame and avoidance strategies (self-distraction, denial, substance use and behavioural disengagement) (Carver 1997). Emotion-focused coping may be maladaptive and lead to depression, anxiety, stress or fear (Folkman 2008; Folkman & Lazarus 1984). Emotion-focused strategies, such as religious beliefs, acceptance and emotional support may facilitate adaptive coping by promoting the use of problem-focused coping strategies (Folkman 2008; Folkman & Lazarus 1984) when stressors are perceived as changeable or manageable. Problem-solving approaches include defining the problem, generating alternative solutions, weighing alternatives and selecting appropriate actions, and lead to favourable outcomes and positive emotions (Folkman 2008; Folkman & Lazarus 1984). Problem-focused coping strategies encompass active coping, instrumental support, positive reframing and planning (Carver 1997). Across different cultural groups and countries, patients with myocardial infarction use mainly emotion-focused coping strategies. The use of emotion-focused strategies has been reported in studies conducted in Iran (Bafghi et al. 2018), Pakistan (Khaliq & Rafiq 2021), China (Du et al. 2020), United Kingdom (Messerli-Bürgy et al. 2015) and Poland (Kroemeke 2016). A Jordanian qualitative study found that participants employed religious and emotional support as emotion-focused coping strategies following myocardial infarction (Ammouri et al. 2017). Problem-focused coping strategies are not primarily used by patients with myocardial infarction (Bafghi et al. 2018). Studies in Pakistan (Khaliq & Rafiq 2021) and China (Du et al. 2020) reported low mean scores for using problem-focused coping by patients with myocardial infarction, while a few studies have identified reported problem-focused coping as a primary strategy (Kroemeke 2016; Messerli-Bürgy et al. 2015). Various factors influence coping strategies adopted by patients with myocardial infarction. Demographics such as age, gender, marital status and education play a significant role. Women are more likely than men to adopt more emotion-focused coping strategies (Celik, Celik & Taskin Yilmaz 2022; Foà, Fuochi & Fruggeri 2015; Włodarczyk 2016). Spiritual coping strategies are used more frequently by men and persons with higher education levels (Khaliq & Rafiq 2021; Son et al. 2016). Older men patients are more likely to use religious and social support coping strategies, but less likely to use active coping or self-blame (Charizopoulou et al. 2015). Greater social support is associated with emotion-focused strategies such as acceptance and religious coping (Fuochi & Foà 2018; Solano-Ruiz et al. 2021), while limited family or social support is associated with more negative coping strategies (Son et al. 2016). Health status, self-care ability and lifestyle also can shape the coping process. Emotion-focused coping strategies, including religious coping, social support and distraction, are mainly used by patients with lower health status and those with severe illness (Charizopoulou et al. 2015). Lower self-perceived health is associated with increased use of emotion-focused and avoidance strategies (Fuochi & Foà 2018). Ability to perform daily activities can promote positive religious coping (Celik et al. 2022). Longer illness duration may limit or reduce the use of coping strategies, including seeking social support and accessing community resources (Son et al. 2016). Smoking status influences a person’s use of active coping and self-blame strategies, particularly in relation to illness severity (Charizopoulou et al. 2015). In Sweden, Schlyter et al. (2016) examined whether coping was related to smoking cessation and found that among 323 post-myocardial infarction patients, non-smokers (*n* = 58) were more likely to adopt problem-focused rather than emotion-focused or distancing coping strategies. Health literacy is the ability to obtain, understand and use health information and medical services to make health-related decisions (Santana et al. 2021). Health literacy comprises three domains, healthcare, disease prevention and health promotion, with four major dimensions: accessing and/or obtaining, understanding, processing and/or appraising and applying and/or using health information (Pelikan et al. 2014). Inadequate health literacy levels have been reported by patients with myocardial infarction from high and low- and middle-income societies (De Melo Ghisi et al. 2018; Zaben & Khalil 2019). Inadequate health literacy levels may vary between countries. In France, a cross-sectional study among 102 patients with myocardial infarction reported that only 21 (20.6%) respondents had inadequate health literacy levels (Perrin et al. 2021). In the USA, a retrospective cohort study estimating health literacy levels of 696 hospitalized patients with myocardial infarction reported that 31% had a below-basic health literacy level (mean score < 185/500) (Bailey et al. 2015). A cross-sectional study in Germany found that 73 (37.4%) of 171 patients with a history of myocardial infarction had insufficient health literacy levels (Dinges et al. 2022). Studies in other countries have, however, reported higher prevalence of inadequate health literacy levels. In Türkiye, in a cross-sectional study, 144 (61%) of 275 patients with coronary artery disease had a limited health literacy level (Ahcioglu & Yilmazel 2022). In China, Fan et al. (2021) reported that 72.7% (*n* = 158) of 217 post-myocardial infarction patients had inadequate health literacy levels. Similarly, in Iran, a prospective cohort study by Mohammadi et al. (2023) reported inadequate or barely adequate health literacy levels among 78% (*n* = 288) of a convenience sample of 366 hospitalised patients with myocardial infarction. Health information is generally accessible; however, the individual’s ability to appraise the quality and usefulness thereof is more difficult for patients with myocardial infarction (Brørs et al. 2022; Perrin et al. 2021). Demographic factors such as age, gender, marital status, urban or rural residence, education and employment status all influence health literacy levels among patients with myocardial infarction (Ahcioglu & Yilmazel 2022; Mohammadi et al. 2023). Age is reported to influence health literacy levels (Brørs et al. 2022; Zaben & Khalil 2019); however, gender as a factor is mixed, as lower health literacy levels have been found in women (Brørs et al. 2022; Perrin et al. 2021) as well as men (Ahcioglu & Yilmazel 2022). Higher health literacy has been found in married and urban-residing patients (Ahcioglu & Yilmazel 2022; Mohammadi et al. 2023). Lower literacy levels were reported in persons with limited education (Brørs et al. 2022; Mohammadi et al. 2023; Zaben & Khalil 2019) and is associated with lower income and unemployment (Ahcioglu & Yilmazel 2022; Perrin et al. 2021; Zaben & Khalil 2019). In Iran, 65.5% of unemployed respondents had inadequate literacy (Mohammadi et al. 2023); however, in Türkiye, a similar country setting, unemployed participants showed higher literacy (Ahcioglu & Yilmazel 2022). Health status and lifestyle habits also influence health literacy level among patients with myocardial infarction. Poor health literacy was more common in persons who perceived themselves as healthy and those who underwent irregular health checks (Ahcioglu & Yilmazel 2022). Brørs et al. (2022) found higher health literacy levels among physically active patients. A significant association has been reported between tobacco and alcohol consumption and lower health literacy levels (Ahcioglu & Yilmazel 2022; Brørs et al. 2022). Jordan, a low-middle income country in the Middle East, has a population of 11 516 000 (Jordanian Department of Statistics 2023). In the most recent statistical reports, high mortality rates because of myocardial infarction have been reported 1104–1336 of 14348–19676 deaths per year between 2008 and 2016 (Jordanian Ministry of Health 2016). Jordan has 119 hospitals, a ratio of 14 beds per 10 000 population. Health services in the country have significant financial constraints, with only 6.2% of the country’s total annual budget allocated to health services (Jordanian Ministry of Health 2024). To minimise the burden on healthcare services, the limited health literacy levels and maladaptive coping strategies of persons with myocardial infarction must be addressed (De Melo Ghisi et al. 2018; Kanejima et al. 2022; Palumbo 2017; Peltzer et al. 2020). Limited published research exists on the coping strategies and health literacy levels of Jordanian patients with myocardial infarction.

### Aim of the study

This study examined the association between commonly used coping strategies and health literacy levels among Jordanian patients with myocardial infarction.

## Methods

### Design

A descriptive, cross-sectional design was used in this study.

### Population and sampling

The population comprised 140 patients with myocardial infarction attending four hospitals in Jordan, including medical wards, cardiac care units and cardiology clinics. The total population was estimated at 750 based on the study of Ashour et al. (2020), with a prevalence of 50%, a degree of precision of 10% and 95% confidence interval. The sample size was calculated at 97 (Dhand & Khatkar 2014), using OpenEpi software, based on previous studies (Hamdan-Mansour et al. 2014; Wångdahl et al. 2014). To reduce selection bias and improve generalisability of results, a stratification process, using hospital bed capacity, was used to determine number of respondents from each hospital. Inclusion criteria were adult Jordanian residents with a history of either recent or prior myocardial infarction event, regardless of whether hospitalisation was because of myocardial infarction or another condition, who could communicate in Arabic, exhibited no obvious cognitive impairment, and were able to recall and describe their myocardial events. Persons who were severely ill because of a recent acute myocardial infarction were excluded. A systematic random sampling process was used. For inpatient respondents, nurses in each unit select every fourth patient (numbered according to date and time of admission). In outpatient settings, the patient presentation time was used to select every fourth patient presenting to the clinic until the desired sample size was obtained. Respondents who met the inclusion criteria were invited to participate in the study.

### Measures

Two scales were used, with the authors’ permission, to collect data.

#### The Arabic version of the Brief COPE ‘AR-BC’ scale

The Brief COPE measures individuals’ coping strategies (Carver 1997). The Arabic version was developed by Hamdan-Mansour et al. (2014). It comprises 14 domains, each consisting of two items. The 28-item four-point Likert scale ranges from 1 ‘I have not been doing this at all’ to 4 ‘I have been doing this a lot’. Scores are calculated based on three coping strategies categories: problem-focused (8 items), emotion-focused (12 items) and avoidant (8 items). Cronbach’s alpha in this study was 0.82.

#### The Arabic version of the 16 items European Health Literacy Survey Questionnaire

The scale (Wångdahl et al. 2014) comprises 16 items taken from the longer European Health Literacy Survey Questionnaire original matrix-related version (HLS-EUQ47) (Sørensen et al. 2013). The instrument measures three health literacy domains, healthcare, disease prevention and health promotion and focuses on four dimensions: accessing or obtaining, understanding, processing or appraising and applying or using health information (Pelikan et al. 2014). Answer categories ‘very easy’, and ‘fairly easy’ score a value of ‘one’ and ‘fairly difficult/very difficult’ or missed items/don’t know score ‘zero’. Scale values are calculated as simple summed scores only for respondents who answer at least 14 items. Scoring ranges from 0 to 16, establishing three levels of health literacy: inadequate (0–8), problematic (9–12) and sufficient (13–16). Cronbach’s alpha in this study was 0.86.

### Ethical considerations

Ethics approval was granted by the Biomedical Science Research Ethics Committee of the University of the Western Cape (Reference BM21/10/41), the Jordanian Ministry of Health and the health facilities. This study adhered to the Protection of Personal Information Act (POPIA) regulations in South Africa (Republic of South Africa 2013) and the Personal Data Protection Law (PDPL) in Jordan (Jordanian Legislation and Opinion Bureau 2023). Measures to protect respondents’ dignity, privacy, welfare, rights, minimise potential harm and maximise benefits included voluntary consent, anonymity and confidentiality through the use of separate sealable envelopes for consent forms and responses. Data management, storage and disposal adhered to the university regulations.

### Data collection

Data were collected between May and October 2022. The researcher provided detailed study information and addressed queries before obtaining consent. Consenting patients received research materials and instructions regarding the instruments. Each set of materials included an Arabic information sheet, informed consent form, survey questionnaire, pen and sealable envelope. Signed consent forms were submitted separately, and completed questionnaires were sealed in envelopes and returned to the researcher.

### Data analysis

Data were analysed using SPSS software (IBM SPSS Statistics for Windows, Version 28.0. Armonk, NY: IBM Corp.). Descriptive statistics, including means, standard deviations, frequencies and percentages, were used to describe demographics and clinical variables. Spearman and Pearson Chi-Square tests were performed to examine associations between respondents’ coping strategies and their health literacy levels, with the significance set at α = 0.05.

## Results

### Sample realisation

The sample comprised 140 respondents, with a mean age of 55.79 years (s.d. = ± 13.26). Most respondents were male, Muslim, living with a spouse, had primary or secondary school education level, unemployed and had valid health insurance. Family members, including sons and daughters, were reported as the primary social supporters (Table 1).

**TABLE 1:** Respondents’ demographics (*N* =140).

Characteristics	Statistics			
	*n*	%	*M*	s.d.
Age (years)	-	-	55.79	13.26
**Gender**				
Male	108	77.10	-	-
Female	32	22.90	-	-
Living with spouse	117	83.60	-	-
Number of family members	-	-	6.28	3.05
**Social supporter (more than one could be selected)**				
No one	38	27.10	-	-
Parents	12	8.60	-	-
Partner	39	27.90	-	-
Family	79	56.40	-	-
Friends	9	6.40	-	-
**Educational level**				
No response	1	-	-	-
None	16	11.40	-	-
School	85	60.70	-	-
Higher education	38	27.10	-	-
**Religion**				
Muslim	139	99.30	-	-
Christian	1	0.70	-	-
**Living area**				
Non-urban	22	15.70	-	-
Urban	118	84.30	-	-
**Current employment status**				
Unemployed	95	67.90	-	-
Employed	45	32.10	-	-
Monthly income	-	-	316.42	273.49
Valid health insurance	110	78.60	-	-

s.d., standard deviation.

### Respondents’ clinical characteristics and lifestyles

The average period since the first myocardial infarction event was 4.86 years (s.d. = ± 6.53). Half of the respondents indicated a history of chronic diseases (*n* = 70), while just over one third (35.7%, *n* = 50) had experienced complications following a myocardial infarction. Twenty per cent (*n* = 28) of the respondents struggled to effectively manage the effects of the myocardial infarction disease and 29 (20.7%) respondents had difficulties with self-care. Over half of the respondents had no exercise plan (66.4%, *n* = 93). Forty per cent (*n* = 56) of respondents were smokers, averaging 30 cigarettes per day. The mean number of years of cigarette use was 31.8 years (s.d. = ± 12.6).

### Coping strategies used by respondents

There was low engagement with coping activities. Emotion-focused coping strategies, 2.54/4 (± 0.53), had the highest mean score. Respondents used mainly emotion-focused strategies, including religious coping (3.15/4 ± 0.93), acceptance (2.95/4 ± 0.86) and emotional support (2.68/4 ± 0.89) but less frequently humour (1.66 ± 0.87. The lowest mean scores 1.83/4 (± 0.43) were for the categories avoidant coping with substance use (1.13/4 ± 0.44) and denial (1.9/4 ± 0.88) (Table 2).

**TABLE 2:** Coping styles and strategies.

Coping category and/or strategies	*M*/4	s.d.
**Emotion-focused**	**2.54**	**0.53**
Religion	3.15	0.93
Acceptance	2.95	0.86
Emotional support	2.68	0.89
Self-blame	2.46	1.09
Venting	2.32	0.93
Humour	1.66	0.87
**Problem-focused**	**2.44**	**0.72**
Positive reframing	2.56	0.87
Planning	2.47	0.97
Active coping	2.38	0.87
Informational or instrumental support	2.36	0.98
**Avoidant coping**	**1.83**	**0.43**
Self-distraction	2.16	0.85
Behavioural disengagement	2.14	0.86
Denial	1.9	0.88
Substance use	1.13	0.44

s.d., standard deviation.

### Respondents’ health literacy levels

Approximately one third (28.6% *n* = 40) of the respondents had inadequate or problematic (32.1% *n* = 45) health literacy levels. The mean health literacy score was 10.87 of a possible 16. In measuring health literacy levels by dimensions, the highest mean score was for understanding and the lowest was for processing information. For health literacy domains, the highest mean score was for healthcare and the lowest for disease prevention (Table 3).

**TABLE 3:** Respondents’ health literacy levels (*N* = 140).

Items	Statistics			
	*M*	s.d.	*n*	%
**Health literacy level/16**	**10.87**	**4.13**	-	-
Sufficient	-	-	55	39.3
Problematic	-	-	45	32.1
Inadequate	-	-	40	28.6
**Understand information/6**	**4.4**	**1.62**	-	-
Understand family members’ or friends’ advice on health/1	0.80	0.40	-	-
Understand doctor’s/pharmacist’s instructions about medicine/1	0.79	0.41	-	-
Understand what the doctor says/1	0.78	0.41	-	-
Understand warnings about behaviour/1	0.73	0.45	-	-
Understand the rationale for health screenings/1	0.66	0.48	-	-
Understand the media information on how to be healthier/1	0.65	0.48	-	-
**Apply information/3**	**2.11**	**0.97**	-	-
Follow medical instructions/1	0.73	0.45	-	-
Use the doctor’s information to make health decisions/1	0.71	0.45	-	-
Decide how to avoid illness based on media information/1	0.67	0.47	-	-
**Obtain/Access information/4**	**2.61**	**1.17**	-	-
Find information on treatments for illnesses/1	0.78	0.42	-	-
Find out where to get professional help when you are ill/1	0.74	0.44	-	-
Find out about good activities for mental well-being/1	0.61	0.49	-	-
Find information on how to manage stress and depression/1	0.50	0.50	-	-
**Process information/3**	**1.75**	**1.09**	-	-
Judge which everyday behaviour is related to health/1	0.64	0.48	-	-
Judge when need to get an opinion from another doctor/1	0.56	0.50	-	-
Judge reliability of the media information on health risks/1	0.55	0.50	-	-
**Health literacy level by domains**				
Healthcare/7	5.09	2.09	-	-
Health promotion/4	2.69	1.24	-	-
Disease prevention/5	3.09	1.60	-	-

s.d., standard deviation.

### Association between coping strategies and health literacy level

No correlations were found between the coping styles or strategies used and respondents’ demographic or clinical characteristics. There were positive associations between health literacy levels and planning, acceptance, active coping, emotional support and positive reframing strategies. Planning had the highest correlation coefficient (*r* = 0.314). A negative correlation was found between venting and health literacy level (*r* = −0.169) (Table 4).

**TABLE 4:** Correlations between health literacy levels and coping strategies.

Coping strategies	Health literacy level
Planning	0.314**
Acceptance	0.273**
Active coping	0.239**
Emotional support	0.175*
Venting	−0.169*
Positive reframing	0.166*

* , Correlation is significant at the 0.05 level (2-tailed);

** , Correlation is significant at the 0.01 level (2-tailed).

## Discussion

Patients who have experienced myocardial infarction are at significant risk of encountering stressors, complications and lengthened hospital stays (AbuRuz, Alaloul & Al-Dweik 2018). Effective coping strategies are essential for facilitating psychological adaptation following myocardial infarction and enhancing adherence to recommended medical management and lifestyle modifications (Lynggaard et al. 2017).

### Coping strategies

Jordanian patients with myocardial infarction exhibited low scores in coping strategies. Low coping strategies scores on the Brief-COPE scale may reflect insufficient coping skills, a lack of perceived stressors or preference not to disclose personal information (Carver 1997). Given that all respondents had experienced a myocardial infarction, a known stressor (Ginzburg et al. 2016; Singh et al. 2017), and had voluntarily participated in the survey, the low coping scores most likely indicate inadequate coping skills. Respondents’ three most frequently employed coping strategies were religion, acceptance and emotional support (all emotion-focused coping mechanisms). These findings align with a qualitative study in Jordan that reported that participants’ main coping strategies after a myocardial infarction event were religious beliefs and practices (Ammouri et al. 2017). Nabolsi and Carson (2011), in a qualitative study of 19 Jordanian men with coronary artery disease, including myocardial infarction, found that participants’ religious beliefs played a significant role in helping them to cope positively with their illness. Respondents in this study mainly employed emotion-focused coping rather than problem-focused strategies. Bafghi et al. (2018) in Iran, in a case-control crossover study of 220 patients with acute myocardial infarction, found that 66.3% (*n* = 146) of the respondents reported using emotion-focused coping strategies, including avoidance behaviours, in reaction to myocardial infarction events. A cross-sectional study in China with involving 326 patients with myocardial infarction found that dysfunctional emotion-focused coping strategies – specifically – avoidance and yielding were most used, with mean scores of 22.6/28 ± 3 and 14.2/20 ± 3, respectively (Du et al. 2020). Religious or spiritual coping can foster positive emotions that enable the patient to cope using problem-focused strategies, thus facilitating adaptation and healthy lifestyles (Folkman 2008). In this study, no significant correlation between coping strategies and respondents’ demographic or clinical characteristics was found. Other studies, have, however, reported that demographics may influence patients’ use of coping strategies (Al Ali & Al Ramamneh 2022; Fuochi & Foà 2018; Khaliq & Rafiq 2021; Liu et al. 2018; Solano-Ruiz et al. 2021; Son et al. 2016). Several studies have reported a correlation with clinical characteristics and adoption of coping strategies by patients with myocardial infarction (Ammouri et al. 2017; Charizopoulou et al. 2015; Fuochi & Foà 2018; Liu et al. 2018; Zaben & Khalil 2019). These differences may be attributed to the premise that the coping process hinges on individual appraisal of stressors, which varies among individuals (Folkman & Lazarus 1984:22–23).

### Health literacy practices

More than half (60%) of respondents in this study exhibited limited health literacy (insufficient and problematic health literacy), a proportion significantly higher than that reported in high-income countries such as France (21%) (Perrin et al. 2021), the USA (31%) (Bailey et al. 2015) and Germany (37.4%) (Dinges et al. 2022). Similar results have been reported in low-middle-income countries such as Türkiye, at 61% (Ahcioglu & Yilmazel 2022) and China, at 73% (Fan et al. 2021). These findings suggest that the low health literacy levels among Jordanian patients with myocardial infarction are primarily attributed to a reduced ability to process health information, also observed by Diederichs et al. (2018) in Germany. This highlights that mere access to health information is insufficient to improve health literacy levels and the importance of assessing patients’ capacity to process health information. Respondents in this study faced challenges in accessing information regarding the management of mental health issues, such as stress and depression, also reported by Diederichs et al. (2018). Limited health literacy affects patients’ ability to cope with stressors and psychological complications, potentially hindering adaptation, reducing treatment adherence and increasing morbidity and mortality rates (Aaby et al. 2017; De Melo Ghisi et al. 2018). No significant correlations between health literacy level and respondents’ demographic or clinical characteristics were found in this study. Demographic factors that may correlate with limited health literacy levels have been reported, such as lower educational levels (Brørs et al. 2022) and poor economic status (Ahcioglu & Yilmazel 2022; Perrin et al. 2021). Health and clinical history-related factors that influence health literacy levels among patients with myocardial infarction are self-care (Zaben & Khalil 2019), lifestyles (Aaby et al. 2017; Brørs et al. 2022) and physical and psychological complications (Ahcioglu & Yilmazel 2022; Brørs et al. 2022).

### Association between coping strategies and health literacy level

In this study, higher health literacy levels were positively associated with the use of problem-focused coping strategies (planning, active coping and positive reframing) and adaptive emotion-focused strategies (emotional support and acceptance) but negatively associated with maladaptive emotion-focused strategies (venting). Two systematic reviews (De Melo Ghisi et al. 2018; Peltzer et al. 2020) reported that higher health literacy levels are fundamental to facilitate adaptive coping, whereas limited health literacy is associated with maladaptive adaptive coping strategies (Aaby et al. 2017). This underscores the importance of assessing and promoting health literacy among patients with myocardial infarction to help them achieve adaptive coping and reduce associated risks.

## Conclusion

Patients with myocardial infarction may exhibit limited health literacy levels, inadequate adaptive coping skills and show a predominant reliance on emotion-focused coping strategies. Enhancing health literacy may play a critical role in improving patients’ adaptation to myocardial infarction, potentially reducing morbidity and mortality and promoting long-term health outcomes following the myocardial infarction. The promotion of adaptive coping among patients with myocardial infarction by enhancing health literacy presents an opportunity to reduce the financial burden of care associated with complications, recurrence and hospital readmissions for patients and healthcare facilities. Health literacy assessment and patients’ use of adaptive coping strategies should be a fundamental to the nursing care of patients with myocardial infarction. Health literacy practices and assessment thereof should be included in nursing education curricula to ensure that nurses compete to perform this important assessment. Further research is needed to determine the causes of low health literacy levels among patients with myocardial infarction.

### Limitations

This study was limited by a small, predominantly male sample with limited education levels, which may restrict the generalisability of the results.
